# The Role of Natural Products in Rheumatoid Arthritis: Current Knowledge of Basic In Vitro and In Vivo Research

**DOI:** 10.3390/antiox10040599

**Published:** 2021-04-13

**Authors:** Georgia-Eirini Deligiannidou, Vasiliki Gougoula, Eugenia Bezirtzoglou, Christos Kontogiorgis, Theodoros K. Constantinides

**Affiliations:** 1 Laboratory of Hygiene and Environmental Protection, Department of Medicine, Democritus University of Thrace, GR-68100 Alexandroupolis, Greece; edeligia@med.duth.gr (G.-E.D.); vasigoug@med.duth.gr (V.G.); empezirt@med.duth.gr (E.B.); tconstan@med.duth.gr (T.K.C.); 2 Institute of Agri-Food and Life Sciences, Hellenic Mediterranean University Research Centre, GR-71410 Heraklion, Greece

**Keywords:** rheumatoid arthritis, antioxidant, natural products, anti-inflammatory, animal studies, cell models

## Abstract

Rheumatoid arthritis (RA) is an autoimmune disorder affecting a vast variety of the population. The onset of RA as well as the development of systematic immunization is affected by both genetic and environmental risk factors. This review aims to point out the role of natural products in the management of RA, focusing on the reports of basic research (in vitro and animal studies) emphasizing the antioxidant and anti-inflammatory properties considered in the field of RA. A systematic screening of the relevant literature was carried out on PubMed, Google Scholar, and Scopus with the following criteria: publication date, 2015–2020; language, English; study design, in vitro or animal models; and the investigation of one or several natural products in the context of RA, including, when available, the molecular mechanisms implicated. A total of 211 papers were initially obtained and screened. In vitro and animal studies referring to 20 natural products and 15 pure compounds were ultimately included in this review. The outcomes of this work provide an overview of the methods employed in basic research over the past five years, with emphasis on the limitations presented, while demonstrating the potential benefits of utilizing natural products in the management of RA as supported by in vitro and animal studies.

## 1. Introduction

Rheumatoid arthritis (RA) is a chronic systematic autoimmune disease [1]. Although the etiology of RA is not clear, there has been significant progress in understanding the mechanisms of the disease, with both genetic predisposition and environmental components involved in its onset and progression, emphasizing the heterogeneity of RA [2,3]. In general, RA is characterized by progressive symmetric arthritis leading to cartilage damage, bone erosion, and could ultimately cause disability [4]. Clinically, the symptoms of RA significantly differ between early stage and insufficiently treated later stages of the disease. Common manifestations include arthralgia, swelling, redness, and limited motion range, while ulnar deviation, subcutaneous nodules, swan neck deformity, and extra-articular symptoms are manifestations related to later stage and/or untreated severe RA [5,6]. Accompanying the disease etiology, a crucial area of research is related to the biological markers (commonly rheumatoid factor (RF) and anticitrullinated protein/peptide antibodies) corresponding to modifications of RA management, based on its early diagnosis, in addition to proper assessment and prediction of disease severity [7,8,9].

The treatment goals are related to sustained remission or low disease activity via reduced joint inflammation and pain, in addition to the prevention of joint destruction and deformity and improvement in joint function [10]. There are multiple medications utilized for the management of RA such as nonsteroidal anti-inflammatory drugs, disease-modifying antirheumatic drugs (DMARDs), and corticosteroids; however, various side effects, as well as toxicity for the kidneys, the liver, and the heart, are commonly reported in several cases [11,12,13]. The 2019 update from the European League Against Rheumatism considered new evidence on RA management, focusing on the efficacy and safety of conventional synthetic (cs) DMARDs (methotrexate (MTX), leflunomide, sulfasalazine), which represent the first line of therapy for RA and glucocorticoids, which are recommended as a bridging therapy when initiating or changing csDMARD therapies. Furthermore, biological DMARDs (tumor necrosis factor (TNF) inhibitors (infliximab adalimumab, etanercept, certolizumab pegol, golimumab), tocilizumab, abatacept, sarilumab, rituximab, and biosimilar DMARDs) and targeted synthetic DMARDs (the Janus kinase inhibitors tofacitinib, baricitinib, upadacitinib filgotinib) have been recommended in cases where poor prognostic factors are present and the treatment target is not achieved with the first csDMARD strategy [14]. It is clear that new evidence regarding disease management is being taken under consideration and helps form more targeted approaches and guidelines.

The role of nutrition has been explored for several years in the context of RA, with most remarks focusing on the potential malnutrition of the patients due to drug–food interactions affecting nutrient absorption, low bioavailability of nutrients, and in several cases lack of appetite, while another field of investigation has been the utilization of natural products in the management of the disease [15]. In this setting, the role of natural products in the onset of the disease, due to their antioxidant and anti-inflammatory properties, as well as its management has been explored and documented [16,17]. Current efforts toward successful RA management often include complementary and alternative medicine, which have gained both the interest of patients and the research community over the past decade [18,19]. It is a mutual observation among patients and health care providers that conventional therapies may be effective; however, they are not free of side effects, especially regarding long-term treatment and particularly when addressing the goal of alleviating the pain [20,21]. Natural products, among other complementary and alternative approaches, constitute an effective option against RA symptoms due to several anti-inflammatory, palliative, and antiarthritic properties [22,23].

In the antioxidant setting of natural products, RA patients may find the use of natural products beneficial. Although there is conflicting evidence of the role of antioxidants in RA, as this field remains poorly explored, the value of antioxidants in fighting inflammation is well-documented, which explains the fact that the antioxidant properties of natural products are commonly evaluated and usually represent the first step of in vitro evaluation before that of the anti-inflammatory properties [24]. Inflammation is described by a variety of cellular activity and molecular reactions, while a typical inflammatory response that does not reach the resolution state contributes to organ dysfunction [25]. Chronic inflammation is related to diverse molecular and cellular processes, depending on the type of inflamed cells and organs [24]. In the case of RA, the onset and development of the disease-related inflammation can be linked to genetic, epigenetic, and environmental factors. Natural products present anti-inflammatory properties through multiple pathways, such as suppression of actuating molecules (e.g., proinflammatory cytokines and chemokines), modulation of anti-inflammatory mediators (e.g., Interleukin (IL)-4, IL-10), regulation of the T-helper type 17 (Th17)/Regulatory T cells (Tregs) balance (the imbalance of Th1 and Th2 immune responses contributes to the pathogenesis of this disease), and modulation of the osteo-immune cross-talk [26,27]. These effects are in turn the outcome of the control of molecular mediators of inflammation such as mitogen-activated protein kinase (MAPK), nuclear factor kappa-light-chain-enhancer of activated B cells (NF-κBs), and signal transducer and activator of transcription 3 (STAT3) by the bioactive constituents of plant-derived or other natural products. Moreover, natural products can modulate the Th17/Treg balance by affecting the amounts of key cytokines (e.g., IL-6, IL-1β, and transforming growth factor-β (TGF-β)) and certain transcription factors such as Interferon Regulatory Factor 4 (IRF-4), STAT3, RAR-related orphan receptor gamma (RORγt), and forkhead box P3 (Foxp3) [28,29,30]. Similarly, acting via certain cytokines (e.g., IL-17) and other mediators such as receptor activator of nuclear factor kappa-B ligand (RANKL), which is responsible for causing chondrocyte degeneration and pathological bone resorption, natural products can influence the T-cell response as well as the osteo-immune cross-talk and bone health [31,32].

In this study, we present an overview of the literature regarding studies of natural products, which have been evaluated for the treatment of RA in the setting of in vitro and in vivo experiments.

## 2. Materials and Methods

For this review, we performed a systematic search of publicly available articles regarding the use of natural products in the context of RA, focusing on the mechanisms involved in the inflammation present in the disease as well as the potential role of antioxidant properties of these products. This work only includes in vitro and animal studies published from 2015 to 2020 in an effort to avoid overlapping with relevant work previously published which may summarize similar findings.

A systematic screening of the relevant literature was carried on PubMed, Google Scholar, and Scopus with the following criteria: publication date, 2015–2020; language, English; study design, in vitro or animal model; and the investigation of one or several natural products in the context of RA, including, when available, the molecular mechanisms implicated. Keywords included in the initial screening included: “natural product”, “herb”, “plant”, extract”, “rheumatoid arthritis”, “arthritis”, “antioxidant”, inflammation”, “animal”, “rat”, “mice”, “remedy”, “mixture”, “cell culture”, “RA-FLS” and “Raw264.7”. During the second search of the literature, a more targeted approach was considered, directly using the names of known natural products or common compounds that were likely included in relevant research in the context of RA. 

A total of 211 papers were initially obtained and screened. The screening process included the following steps: (1) title and abstract screening for relevance, (2) availability of the original text in English and (3) full-text screening to ensure the suitability of the results for the purposes of this review. Through this process, a number of articles were excluded due to non-English text availability, while smaller numbers were excluded due to relevance with the target of this work or lack of fully available results (Table 1).

In vitro and animal studies referring to 20 natural products and 14 pure compounds were ultimately included in this review. All eligible papers were screened, distributed as per the study design (in vitro and animal studies), and separated into two groups (natural products referring to all forms assessing the effects of a naturally occurring product as a whole and isolated natural compounds). Several components have presented significant beneficial properties.

## 3. Results

### 3.1. Natural Products

#### 3.1.1. Date (*Phoenix dactylifera* L.)

Date (*Phoenix dactylifera* L.) seeds are a known traditional Moroccan remedy against pathological conditions involving inflammation, such as RA [33]. Date seeds are rich in dietary fiber, phenolics, and antioxidants while their protein contains the majority of essential amino acids [34]. An interesting study on the methanol extracts of different date seed varieties (namely: Boufgous, Bousthammi, Jihl, and Majhoul) aimed to evaluate their anti-inflammatory effects, as reflected via membrane stabilization activity, suppression of protein denaturation, and nitric oxide (NO) radical scavenging activity (IC_50_ is reported in all cases). In all cases, 30 g of pulverized date seeds were used for the extraction. The results of this research showed that Boufgous seeds had higher rates of membrane stabilization activity (241.65 ± 6.69 mg/mL) and relatively high outcomes in terms of the inhibition of protein denaturation and NO radical scavenging activity (167.32 ± 5.82 mg/mL and 144.45 ± 7.63 mg/mL, respectively) compared to Trolox, which was used as a reference standard. Majhoul seeds had the highest outcomes in terms of the inhibition of protein denaturation and NO radical scavenging activity (193.71 ± 7.25 mg/mL and 163.63 ± 6.39 mg/mL, respectively), although their results for membrane stabilization were slightly lower than those of the Boufgous seeds (209.38 ± 9.01 mg/mL). Interestingly, these outcomes were related in the article to the phenolic and flavonoid contents of each product as an indicative factor of their antioxidant and anti-inflammatory activity [33]. The research was further carried in a collagen-induced arthritis (CIA) animal model in which, in relation to the previously reported outcomes, date seeds (at a dose of 30 mg/kg body weight (b.wt.)) exhibited significant ability to reduce carrageenan and croton oil-induced paw and ear edema, respectively, as compared to indomethacin (10 mg/kg b.wt.). More specifically, regarding the carrageenan-induced paw edema, Bousthammi and Jihl varieties showed 76.27% and 87.28% reductions, respectively, while the Boufgous and Majhoul varieties resulted in reductions of over 40%. Similarly, croton oil-induced ear edema had in all cases over 50% reduction, reaching up to 77.17% for the Bousthammi seeds, also compared to indomethacin (95.11%) [33]. 

#### 3.1.2. Wild Pomegranate (*Punica granatum* Linn.)

*Punica granatum* has been used as a traditional medicine for the treatment of various conditions including pain and inflammation [35,36]. As described in a recently conducted study in the setting of lipopolysaccharide (LPS)-induced RAW 264.7 macrophages, pomegranate has shown potential NO inhibition, as well as decreased paw edema in carrageenan-induced mice after administration of 100 mg/kg [36]. An interesting study was conducted to evaluate the antiarthritic activity of butanol fraction (administered at doses of 50 and 75 mg/kg body weight) of *Punica granatum* Linn. rind methanolic extract versus dexamethasone (5 mg/kg) against Freund’s complete adjuvant (FCA)-induced arthritis in rats. The results of this study showed dose-dependent effects on biophysical (body weight, paw volume, arthritic score, and joint diameter) and hematological parameters (such as red and white blood cell counts, Erythrocyte Sedimentation Rate (ESR) and Hemoglobin (Hb) concentration), while active phytochemicals such as phenolic compounds, iridoid glycosides, and flavonoids were underlined as the source of the antiarthritic potential of the butanol fraction of *Punica granatum* Linn. rind extract [37].

It is worth noting that NO inhibitory effects of Punicalagin, Punicalin, Strictinin A, and Granatin B (hydrolysable tannins found in this natural product) were measured in LPS-induced RAW 264.7 macrophage cells showing inducible NO synthetase (iNOS) activities of 15.7 ± 0.4, 46.7 ± 7.0, 25.4 ± 2.4, and 41.7 ± 0.7, respectively (results presented as NO inhibition % for each compound). Although the IC_50_ values (mM) for each of these compounds regarding NO inhibition was relatively high (69.8, 78.6, 63.1, and 33.6 mM, respectively), this could also explain the reported levels of cytotoxicity (%) (35.5 ± 0.7, 67.6 ± 1.4, 45.8 ± 1.1, and 41.7 ± 0.7, respectively). However, from further investigation using lower concentrations (12.5–100 μΜ) and although cytotoxicity was still exhibited at 100 μM, dose-dependent inhibitory effects of the iNOS expression were found, which were also related to the time of the reaction showing significant iNOS inhibition at 8 h as well as 18 h [37].

Pomegranate juice is one of the natural products which have also led to promising outcomes in the setting of clinical trials for the management of RA symptoms, which can also be related to the polyphenolic compounds that exert antioxidant and anti-inflammatory activities. Pomegranate extract (250 mg/capsule) has been investigated and compared to cellulose (250 mg/capsule) in 55 RA patients in an 8-week trial. The results demonstrated reductions in major targets of RA management such as swelling and tenderness as well as morning stiffness and pain compared with the placebo group. Additionally, markers such as ESR levels, which have also been investigated in animal studies, have also shown a reduction in the clinical setting, although others such as mean matrix metalloproteinase—3 (MMP3) or C-Reactive protein (CRP) presented no substantial differences between the studied groups [38].

#### 3.1.3. Ribes (*Ribes orientale*, *Ribes alpestre* Decne)

Ribes is a genus comprised of about 200 known species and the only genus in the family Grossulariaceae, which originates from many parts of the Mediterranean area as well as most of Asia [39,40]. The roots of *Ribes orientale* aqueous ethanolic extract (30:70), aqueous and n-butanol fractions at the effective dose of 200 mg/kg (initial screening included doses of 50, 100, and 200 mg/kg b.wt.) were evaluated in treating RA in Sprague–Dawley rats (FCA model) and compared to piroxicam (10 mg/kg b.wt.). The interventions were orally administered 30 min before adjuvant injection from the beginning of the study and until 28 days after the injection. Paw volume/diameter was dose-dependently decreased by the intervention, which also suppressed paw swelling. Additionally, the interventions successfully downregulated gene expression levels of *TNF-α*, Cyclooxygenase type 2 (*COX-2*), *IL-6*, *NF-κB*, *IL-1β*, and Prostaglandin E2 (*PGE*_2_) and upregulated those of *IL-4* and *IL-10* compared with FCA control rats [41]. Similar results were obtained in the study of *Ribes alpestre* Decne, which has been also commonly used in the treatment of joint complaints. In the study of 28-day supplementation with aqueous ethanol extract or n-butanol and aqueous fractions at a 200 mg/kg oral dose in Sprague–Dawley rats (FCA model), paw volume and thickness and arthritic score were scientifically reduced by the intervention. Notably, on day 28, paw volume was decreased at approximately 81%, 61%, and 76% for the aqueous ethanol extract or n-butanol and aqueous fractions, respectively, with similar rates reported for paw diameter. Additionally, downregulation of proinflammatory cytokines IL-1β, TNF-α, IL-6, COX-2, PGE_2,_ and NF-κB was also observed in this study. It is worth mentioning that NF-κB, TNF-α, and IL-1β fold change rates were similar, if not the same, in the cases of piroxicam, aqueous ethanol extract, and aqueous fraction compared to the control group [42]. It should be noted that previous research has indicated the beneficial properties of anthocyanins and proanthocyanidins (the better-known phytochemicals found in Ribes) in conditions involving inflammation such as RA among others [43,44]. These effects will be further discussed later in this review. 

Additionally, regarding the antioxidant properties of plant extract and its fractions, free radical scavenging activity was measured against the 1,1-diphenyl-1-picryl-hydrazyl (DPPH) radical using solutions of various concentrations (50–6400 μg/mL) and ascorbic acid as a reference standard. This study showed that the ethanolic (70%) plant extract exhibited 84.53% inhibition of oxidation at 6400 μg/mL, while butanol and aqueous fraction showed 74.07% and 85.74% DPPH radical scavenging potentials at maximum concentration. Similar results were obtained via the ferric reducing antioxidant power (FRAP) protocol, showing a significant and concentration-dependent (50–6400 μg/mL) increase in reducing power of aqueous ethanol extract and its fractions, with higher results observed in the plant extract at 6400 μg/mL, followed by the aqueous and butanol fractions at the same concentration [43,44].

#### 3.1.4. Southern Dewdrop Grass (*Circaea mollis* Sieb. and Zucc)

*Circaea mollis* Sieb. and Zucc. is the scientific name of Southern Dewdrop Grass originally written as “南方露珠草”, a natural product known in traditional Chinese medicine. This plant has been investigated for the treatment of joint swelling and pain in RA in the form of its ethanol extract in an animal study. The effects of the supplementation with various doses of the ethanolic extract of the plant were evaluated using dimethyl benzene (induction of inflammatory swelling in the ear), hot-plate (induction of pain), and FCA models. The selected doses of 170, 680, and 1350 mg/kg (delivered daily for a week) successfully inhibited the induced swelling (at least 3 mg reduction in swelling) and the high dose resulted in effects similar to the positive control (aspirin 35 mg/kg) (*p* < 0.01). Similar results were observed in the evaluation of time of reaction to pain induced by the hot-plate. In this case, the reaction time was successfully prolonged after treatment, mostly in the groups of 680 and 1350 mg/kg. A dose-dependent effect on both paw swelling and arthritis index was observed after FCA-induced arthritis for both selected doses (470 and 940 mg/kg). However, it is highlighted that the timing of the effects differed 15 days between the low and the high doses. Additionally, the treatment downregulated serum TNF-α and IL-1β and increased the production of serum IL-10 in FCA-induced rats [45]. 

#### 3.1.5. Bailari (*Clematis orientalis* Linn)

*Clematis* is a botanical source of various pharmaceutically active components, especially pentacyclic triterpenoid saponin, with reported anti-inflammatory and antioxidant properties [46]. A recent animal study evaluated the aqueous ethanolic extract and fractions (butanol, hexane, and aqueous) in conditions of FCA immunization. As observed, the oral administration of 200 mg/kg for 28 days, when compared to piroxicam (10 mg/kg/day), was able to adequately reduce paw volume (higher than 60% for all fractions on day 28, when piroxicam resulted in 77.18%) and similar results were observed in response to thickness. Additionally, all treatment groups were able to suppress the expression levels of IL-1β, TNF-α, IL-6, COX-2, and NF-κΒ with the most distinct observations in the case of the aqueous ethanol extract, the aqueous fraction, and the butanol fraction as compared to piroxicam treatment. Finally, different treatments also modulated the levels of TNF-α and PGE_2_ in which cases the butanol and aqueous fractions had similar results [47]. It is worth noting that investigations of this natural product in the animal setting have also demonstrated promising outcomes in terms of the management of postmenopausal osteoporosis, which is also related to the antioxidant effects of several phytochemicals [48].

#### 3.1.6. Fern (*Matteuccia struthiopteris*, *Osmunda japonica*, *Matteuccia orientalis*, *Pteridium aquilinum*)

Many fern species are used in traditional medicine covering antibacterial, gastric, and renal infection treatment, pain killer (against headaches, stomachaches, gastrointestinal aches), diuretic, and anti-inflammatory activity [49]. Edible ferns are some of the most important wild vegetables in China as a recent study evaluated fern extracts from *Matteuccia struthiopteris*, *Osmunda japonica*, *Matteuccia orientalis,* and *Pteridium aquilinum* in the context of the proinflammatory gene expressions of IL-1β and IL-6. All samples were evaluated in RAW264.7 cells compared to LPS and reported to dose-dependently (doses evaluated: 2.5, 5, 10, 20, 40, 80, and 160 mg/mL) decrease the levels of IL-1β gene expression, while the roots of *O. japonica* and the young fronds of *M. orientalis* seemed to have the leading activity toward this effect. Regarding the antioxidant activity of these natural products, Trolox equivalent antioxidant capacity (TEAC) assay presented in percentage of ABTS radical inhibition and Trolox equivalents (µg/mL), as well as DPPH antioxidant assay, were reported. All the tested natural products were examined in concentrations of 5, 50 and 100 μg/mL. Results exhibited concentration dependency for all assays. Notably, the ABTS assay resulted in higher inhibition percentages for all samples, reaching over 95% inhibition at 100 μg/mL and over 65% inhibition at 50 μg/mL. In a similar way, DPPH results were over 68% for the *O. japonica* roots and rhizome extracts at concentrations of 50 or 100 μg/mL (68.8 ± 0.5 and 71.6 ± 0.6, respectively), followed by the young fronds of *M. orientalis* at concentrations of 50 or 100μg/mL (45.2 ± 1.8 and 65.6 ± 1.7, respectively). These results combined with the evaluation of proinflammatory gene expression highlight a potential connection and importance of antioxidants at the cellular level [50]. Similar results have also been reported on *Davallia formosana*, which also belongs to the family of ferns. However, in the latter study, the investigation was targeting epicatechin-3-*O*-β-d-allopyranoside, which is a component of the plant, and the study is discussed again later in this review. On this basis, these products may also be considered as potential agents against inflammation related to RA.

#### 3.1.7. Schefflera (*Schefflera octophylla* (Lour.) Harms)

*Schefflera octophylla* (Lour.) Harms, a kind of traditional Chinese medicine (TCM) mainly distributed in Southeast Asia, is commonly used for analgesic, fever, anti-inflammatory, rheumatism, and hemostasis therapies [51]. An early study evaluating the ethanol extract and its chloroform active fraction (one of the five polar fractions examined in this study) reported on inhibited ear edema induced by xylene (as compared to indomethacin (10 mg/kg)), and increased pain threshold in hot-plate test in 2 h (extract doses examined in the hot-plate test were: 300, 600 and 1200 mg/kg b.wt.). Notably, no significant dose-dependency was observed in the outcomes of xylene-induced edema, while in all treatments of 300, 600, and 1200 mg/kg the pain inhibition rates were 35.45%, 50.33%, and 62.14%, respectively. Similarly, the examined extracts and fractions, exhibited inhibitory effects on paw swelling and pain in the setting of FCA rats, although the interventions did not produce the same outcomes as the positive control (leflunomide (6 mg/kg)). Additionally, both ethanol extract (600 mg/kg) and chloroform fraction (300 mg/kg) resulted in lower levels of TNF-α, IL-1β, and IL-6 but not significantly lower levels of RF in serum compared to the control group, while, in this case, dose-dependency of the outcomes was also not reported and none of the treatment groups had the same outcomes as the positive control. Finally, histopathological observation demonstrated that the 300 and 600 mg/kg of the ethanol extracts, 150 and 300 mg/kg of the chloroform fractions, and the leflunomide treatment inhibited synovial hyperplasia and inflammatory cell infiltration [52].

#### 3.1.8. Gerard’s Jointfir (*Ephedra gerardiana*)

Endemic to the mountains of Afghanistan, Bhutan, northern India, Nepal, Pakistan, Sikkim, Tajikistan, and Tibet, the whole plant, roots and stems of *Ephedra gerardiana* (Family *Ephedraceae*) have long been used as folk remedies with documented antioxidant and antimicrobial activities [53]. The aerial parts of *Ephedra gerardiana* were evaluated in an FCA-induced arthritis rat model, exploring the effects of a 28-day supplementation (200 mg/kg) with the aqueous ethanolic extract and its aqueous, n-butanol and ethyl acetate fractions, as compared to piroxicam (10 mg/kg). As reported in this study, the extract and fractions (in particular the aqueous fraction) significantly suppressed paw swelling (paw volume inhibition percentage for the piroxicam group was approximately 65%, while ethanol extract and aqueous and butanol fractions presented inhibition rates of approximately 70% on day 28) and arthritic score (suppression levels were more obvious after day 14 and were mostly related to the supplementation groups of ethanol extract, aqueous and butanol fractions). In a similar way, all treatments were able to attenuate the production of PGE_2_, COX-2, TNF-α, IL-1β, NF-κB, and IL-6, while the treatments of ethanol extract, aqueous, and butanol fractions had the more obvious effects in most cases. Particularly in the cases of COX-2, NF-κB, and IL-6, all treatment groups had lower rates compared to piroxicam, while the ethanol extract and aqueous fraction groups had lower rates of TNF-α and IL-1β as compared to piroxicam [54].

The antioxidant potential of *Ephedra gerardiana* aqueous ethanolic extract and fractions was evaluated by 2,2-diphenyl-1-picrylhydrazyl (DPPH) radical scavenging assay at different concentrations (6400, 3200, 1600, 800, 400, 200, 100, 50 µg/mL). The plant extract and fractions exhibited a noteworthy (*p* < 0.001) free radical scavenging potential that was concentration-dependent (50 to 6400 µg/mL), while the maximum scavenging ability at 6400 µg/mL was by crude extract (84.1%) and, amongst the fractions, aqueous fraction (77.6%) [54].

#### 3.1.9. Green Tea (*Camellia sinensis* (L.))

Green tea is one of the most popular natural products consumed worldwide, known and studied for its many beneficial health effects due to a variety of bioactive agents found mostly in the leaves of *Camellia sinensis* [55,56,57]. It is well-documented in various animal models that polyphenolic compounds (especially catechins), also found in green tea, can reduce inflammation in the context of inflammatory arthritis. Considering osteoarthritic and rheumatoid cartilage, the protective effect of such compounds on joint tissues has also been discussed due to the inhibition of proteoglycan and type II collagen breakdown [58]. Similarly, a recent study has highlighted the anti-inflammatory effects of epigallocatechin-3-gallate found in green tea [59]. Additionally, considering the compounds implicated in the therapeutic effects of *Camellia sinensis* (among other plants), strong molecular interactions of epigallocatechin gallate at the potential ligand binding sites of TNF-α and IL-1 were also demonstrated, thus supporting the inhibitory effects against the proinflammatory cytokines TNF-α and IL-1 [60]. Investigating the effects of aqueous extracts from dried leaves of *Camellia sinensis* (L.), a recent study employed oral doses of 50, 100, 200, and 400 mg/kg/body in a CIA model. The antiarthritic dose of 400 mg/kg/body was selected based on previous experiments conducted in this study and resulted in alleviation of tissue swelling, joint deformity, neutrophils infiltration, and pannus formation in the treatment group, as described via radiological and histological assessments (60% efficacy after almost a month) compared to CIA [61]. Investigating the antioxidant properties of *Camelia sinensis* (L.), joint tissues from the sacrificed animals were collected and properly handled in order to evaluate the NO radical scavenging activity. Results of this study showed that NO level was significantly (*p* < 0.01) higher (52%) in CIA group as compared to control group animals. Treatment with *Camelia sinensis* showed a significant (*p* < 0.05) decrease (45%) in the level of ΝO in the CIA and *Camelia sinensis* groups as compared to CIA group, as well as a significant (*p* < 0.05) change compared to control group [61].

#### 3.1.10. Granule (*Bawei Longzuan*)

*Bawei Longzuan* granule (also found in the literature as Ba-Wei-Long-Zuan granule) is a representative Zhuang medicine preparation used for the treatment of RA [62]. A recent study showed that oral administration of 1.25, 2.5, and 5 g/kg for 30 days successfully modulated paw swelling in CIA rats; notably, the higher dose resulted in a rapid decrease after 15 days, which was also slightly observed for the medium dose but not for the lower dose. However, the outcomes were not comparable to those of dexamethasone although paw swelling in all groups was lower than the CIA group. In addition, all treatments were able to decrease the arthritis score by almost half (with significant changes being observed after 20 days of treatment) and to downregulate serum levels of IL-1β, TNF-α, IL-6, and IFN-γ (also in a dose-dependent manner, with the high dose being comparable to dexamethasone in some cases (*p* < 0.01)). It should be noted that the identification of compounds of this natural product in this study presents us with several known constituents such as Sinomenine, Hesperidin, Nitidine, Nobiletin, and others with reported anti-inflammatory activity, some of which are further examined in this review [63].

#### 3.1.11. Chinaroot, Tufuling (土茯苓) or Sarsaparilla (*Smilax glabra*) 

Focusing on the polysaccharides of the *Smilax glabra* rhizomes, a recent study investigated two polysaccharide fractions, SGP-1 and SGP-2, isolated from the rhizomes of *S. glabra*. The main monosaccharide compositions in both cases were galactose and rhamnose (2.5:1). In LPS-induced RAW 264.7 cells, both SGP-1 and SGP-2 have significantly modulated TNF-α and IL-6 expression levels and repressed the extracellular signal-regulated kinase (ERK) and c-Jun NH2-terminal kinase (JNK). Additionally, investigating the NO inhibitory effects of this product, this study demonstrated that when RAW 264.7 cells were treated with different doses of SGP-1 or SGP-2 (25, 50, 100, and 200 μg/mL) together with LPS for 24 h, significant concentration-dependent inhibition of NO production was detected. There was a highly significant decrease in the NO production when compared with the LPS-alone group (*p* < 0.01). To establish an association between the reductions in LPS-induced NO, TNF-α and IL-6 and transcriptional inhibition, the mRNA levels of iNOS were measured, showing that the mRNA expression levels of iNOS were decreased in a dose-dependent manner by both SGP-1 and SGP-2, while the first also exhibited a notably downregulation effect on LPS-induced mRNA expression of TNF-α [64]. More recent studies have been focused on the identification of the phenolic compounds of *Smilax glabra,* showing promising results regarding their anti-inflammatory properties based on moderate IL-1 expression inhibitory activities on LPS-induced Th-1 cells and NF-κB induction [65,66].

#### 3.1.12. Liana “Tondin” (*Paullinia pinnata* L.)

*Paullinia pinnata* L. (Sapindaceae) is an African woody vine widely used in Ghana and is traditionally used, inter alia, for the treatment of itch- and pain-related conditions such as RA [67,68,69]. An interesting study examined the anti-inflammatory and analgesic effects of aqueous and methanol extracts from *Paullinia pinnata* leaves. This study considered both in vitro and in vivo evaluation of the extracts at different concentrations in each case (10, 30, and 100 μg/mL for the in vitro study and 100, 200, and 300 mg/kg/day administered orally for the in vivo study). Regarding the animal model, FCA rats were evaluated during the 14 days of treatment, showing a significant decrease in the inflammation located in the ankle and paw in addition to the reduction in pain sensation in both sites. Reporting on the effects of both interventions (aqueous and methanolic extracts in the concentrations mentioned), non-stimulated and LPS-stimulated (8 h exposure) RAW264.7 cells, *P. pinnata* extracts effectively recused NO production by 47–88% in the stimulated cells, unlike the case of non-stimulated cells, while both treatments were able to modulate TNF-α (35–68%) and IL-1β (31–36%) production in LPS-stimulated macrophages. It should be mentioned that both plant extracts presented no cytotoxic effects, while the methanol extract also a concentration-dependent affinity for Sigma 2 receptors with an IC_50_ of 50 μg/mL [70].

#### 3.1.13. Salt Cedar (*Tamarix ramosissima* Ledeb)

*Tamarix ramosissima* Ledeb is a folk herbal remedy used for RA management in northwest China. Two studies investigating the compounds of this plant have also reported the viability of RA fibroblast-like synoviocytes (FLSs), via MTT (3-(4,5-dimethylthiazol-2-yl)-2,5-diphenyltetrazolium bromide) assay, and proapoptotic effects of the newly and previously isolated compounds. In the form of aqueous/ethanol extract, *T. ramosissima,* inter alia, remarkably induced cellular apoptosis of RA-FLS, increased the activated caspase-3/7 levels (caspase activity assays are related to the detection of early apoptosis), and significantly increased sub-G1 fraction in the cell cycle [71,72]. It is worth noting that the role of apoptosis has been previously described within the context of RA. In short, RA-FLSs are resistant to apoptosis induced by the apoptotic stimulus signals, while multiple mechanisms, highly activated in the RA joint, may contribute to the reduced apoptosis. Pathways such as the NF-κB, phosphatidylinositol 3-kinase/Akt-1, and signal transducer and activator of transcription-3 pathways are also related to the expression of a variety of antiapoptotic molecules in addition to their modulating effects on inflammation [73].

#### 3.1.14. Thunder God Vine, Thunder Duke Vine or Léi Gōng Téng (*Tripterygium wilfordii*)

Several advantages related to this traditional Chinese herb have been previously reported, including properties of decreasing ESR, RF, C-Reactive Protein (CRP), and risk of adverse events related to RA [74]. Focusing on the effects of this product on dipeptidyl peptidase I (DPPI), a lysosomal cysteine protease derived from granule immune cells, a study employed in vivo and in vitro assays for investigation. In the setting of CIA rats, *Tripterygium wilfordii* was investigated as a single preparation at two different doses (the low dose was 2.5 mg/100 g body-weight and high dose was 5 mg/100 g body-weight), while triptolide, a known compound of this plant, was also employed as a separate intervention group (4 μg/100 g body-weight) in addition to the control and untreated CIA rat group. As reported in this study, treated CIA rats had better outcomes regarding bone erosion, paw swelling, and total protein concentration in the SFs (total protein concentrations were: 526 ± 145, 1652 ± 32.8, and 932 ± 105 for the control group, the untreated group, and the treated group, respectively). In particular, the treatment of 5 mg/mL was able to improve paw swollen thickness, especially after the 30th day as compared to the untreated group (treatment group swollen thickness mean ± SEM (cm): 0.44 ± 0.03 and 0.39 ± 0.01, untreated group swollen thickness mean ± SEM (cm): 0.84 ± 0.02 and 0.83 ± 0.01, on days 31 and 33, respectively). Furthermore, the treatment of 5 mg/100 g b.wt. inhibited the activity of DPPI in the serum of CIA rats at a rate of approximately 58% compared to the untreated group, while it should be mentioned that the treatment with triptolide had no significant effect in this case as compared to the untreated CIA mice [75]. Further evaluation focusing on triptolide is presented later in this review.

*Tripterygium wilfordii* Hook F. (20 mg three times a day) has also been investigated in the context of efficacy and safety in a clinical trial (24 weeks) involving 207 patients with active rheumatoid arthritis and compared with methotrexate (12.5 mg once a week) treatment as well as a combined treatment. This study underlines a series of positive outcomes of efficiency (similar ones were also reported in the animal model) such as the American College of Rheumatology 20/50/70 (ACR20, ACR50, ACR70) response, remission rate and low disease activity rate as well as significant improvements in the health assessment questionnaire and 36-item short-form health survey questionnaire scores. However, side effects, namely the development of irregular menstruation, are also considered and reported for all treatment groups [76]. In the 2-year follow-up of the “Comparison of Tripterygium wilfordii Hook F. with methotrexate in the Treatment of Active Rheumatoid Arthritis” (TRIFRA) study, which investigated *Tripterygium wilfordii* Hook F. compared or combined with methotrexate in controlling the manifestations in patients with DMARD-naïve active RA, it is demonstrated that *Tripterygium wilfordii* Hook F. monotherapy was not inferior to MTX monotherapy in controlling disease activity and retarding radiological progression [77]. It is worth noting that treatment with *Tripterygium wilfordii* Hook F. in the setting of RA has also been investigated further in the context of identifying the predictors to its response in a two-stage trial involving overall more than 300 patients with active RA. In this study, *Tripterygium wilfordii* Hook F. therapy was compared to methotrexate and sulfasalazine combination therapy in a setting of patients classified into predictor positive and predictor negative groups. Results of these efforts have led to the identification of five predictors (diuresis, excessive sweating, night sweats for positive; and yellow tongue-coating, thermalgia in the joints for negative), which further highlights that natural products can be both efficient and effective [78]. 

#### 3.1.15. Pei Lan (*Eupatorium japonicum* Thunb.)

The roles of increased proliferation and insufficient apoptosis in the RA joint, which contribute to synovial membrane hyperplasia, have been previously described in this review, while several investigations are focusing on the modulating effects of natural products as well as reactive oxygen species (ROS) in this context. *Eupatorium japonicum* Thunb (also reported as *Eupatorium chinense* L.) is a widely used folk herb in many traditional Chinese herbal formulas [79]. The extract of *Eupatorium japonicum* Thunb was investigated among others in a study. Employing RA-FLS (MH7A) cells to investigate viability (via the MTT assay), the study reports affective modulation by the treatment (37.5 μg/mL for 6 h) through the induction of ROS-mediated apoptosis (the intensity of intracellular ROS was evaluated via flow cytometry and fluorescence microscopy utilizing treatment with 20 μM DCF-DA). In addition, the intervention dose-dependently inhibited the TNF-induced expression of IL-1β and the transcription of MMP-9 through the inhibition of NF-κB and p38 activation [80].

#### 3.1.16. Herbal Mixtures and Formulas

The role of mixtures has been also highlighted in the literature with various examples, mostly addressing their general capacity to effectively fight inflammation [81,82]. In a recent study, a herbal preparation consisting of Aglianico grape pomace, propolis, pomegranate peel, and extracts (1:4:1) was evaluated for its effect on a murine CIA model. The concept was to evaluate the protective effect of the mixture against RA while also studing the effect of the time-of-treatment in the CIA model. Thus the treatment groups differed in the time of the mixture introduction after CIA induction (namely, aside from the untreated group (Group 1: non-induced control group, treated with vehicle) and the positive control group (Group 2, CIA-induced, treated with 50 μL of bovine type II collagen emulsified in CFA on day +1 and +21), Group 3 received the mixture (propolis (100 mg/kg), pomegranate extract (25 mg/kg) and pomace extract (25 mg/kg)), right after CIA induction and Group 4 received it 3 weeks later). In this study, the early treatment appeared to be beneficial as it resulted in a delayed onset of the disease (CIA onset on day 35) and alleviated the severity of the clinical symptoms, while it was also associated with a reduction in serum levels of the proinflammatory cytocides IL-17-, IL-1b-, and IL-17-triggering cytokines, which are particularly implicated in the pathogenesis of RA [83]. 

*Bolbostemma paniculatum* (Maxim.) Franquet (Tubeimu) and *Smilax glabra* Roxb. (Tufuling) are also reported to be used as complementary medicine in TCM. In a study investigating both the compound characterization (focusing mostly on the quantification of Astilbin and Tubeimoside I in the ethanolic extract of the plant mixture) and the anti-inflammatory activity of this product within the frame of RA, a carrageenan-induced paw edema rat model was employed. The effects of the ethanolic extract of a mixed preparation of rhizomes of *Smilax glabra* Roxb. (125 g) and bulbs of *Bolbostemma paniculatum* (Maxim.) Franquet (375 g) were examined against the dexamethasone and the control group in rats. Oral administration of daily treatments (mixed preparation: 346.5 mg/kg or dexamethasone: 0.5906 mg/kg or sodium cellulose glycolate (0.5%): control) was carried for a week. As reported, the treatment inhibited the carrageenan-induced paw edema by approximately 10% and 20% after 3 and 5 h of injection, respectively, compared to the untreated group and successfully downregulated the levels of proinflammatory cytocides IL-1β, IL-6 and TNF-α (*p* < 0.01 compared to the untreated group) [84].

### 3.2. Natural Compounds

#### 3.2.1. Polyphenols

##### Resveratrol (Stilbenes)

**Figure 1:**
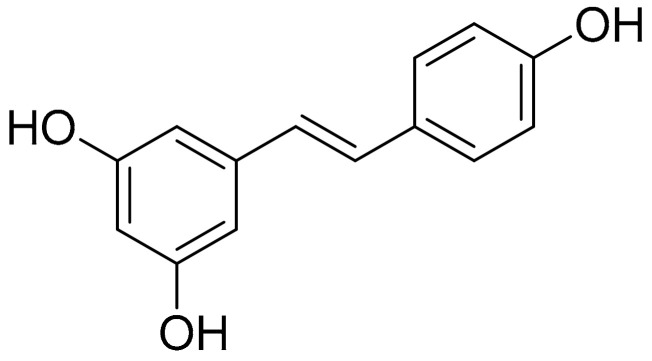
Resveratrol structure.

Resveratrol (Figure 1) is a well-known and thoroughly studied stilbene derived from stilbenoid biosynthesis. This phytoalexin polyphenolic compound, mostly recognized for its antioxidant profile, is commonly abundant in various natural products, such as grapes, berries, and peanuts [85]. As previously reported, there is compelling evidence on the beneficial effects of this plant-derived constituent on autoimmune diseases, while a correlation has been described between the disease activity of RA and the presence of oxidative stress, especially oxidative DNA damage. In the case of RA, the benefits of resveratrol are established, inter alia, on the basis of proinflammatory cytokine (IFN-γ, TNF-α, IL-6, IL-1, and IL-4) modulation, as well as MMPs and RANKL inhibition [86]. A study demonstrated that the upregulation of Sirt1 by resveratrol suppressed the Bradykinin-induced COX-2/PGE_2_ production through inhibiting the interactions of AP-1 and NF-κB with COX-2 promoter in RA synovial fibroblasts, while resveratrol also inhibited the phosphorylation and acetylation of p65, and reduced the binding to the COX-2 promoter, thereby attenuating COX-2 expression [87]. Similar results were observed in a study employing an acute model of antigen-induced arthritis in rats. As reported, pretreatment with resveratrol (12.5 mg/kg daily for 2 months before antigen-induced arthritis (AIA) induction) led to a significant reduction in knee swelling, which was documented after the continuous oral administration of resveratrol 2 days after the injection [88].

Furthermore, in the setting of acute antigen-induced arthritis (AIA) (including control, AIA, and resveratrol-treated (12.5 mg/kg/day) AIA groups), resveratrol was given orally 2 months before AIA induction until 2 days after injection. As reported, the treatment significantly reduced the histological score of synovial tissue and increased the expression of LC3 signals in the AIA synovial membranes. Furthermore, the treatment also mitigated p65 expression and decreased articular cartilage degradation. From the antioxidant protection perspective, it has been documented that resveratrol modulates oxidative DNA damage and peroxidase activity in synovial tissue. In relation to this, a study examining the potential of oral administration of resveratrol to induce changes in the levels of synovial 8-oxo-dG, a major biomarker of oxidative DNA damage, also employed an AIA model including the untreated and healthy group, the untreated-AIA group and the treated (12.5mg/kg of resveratrol) AIA group of rats. The results demonstrated a reduction in 8-oxo-dG expression in the synovial tissue from the treatment group compared to the AIA group, while the resveratrol treatment was also related to a significant enhancement in peroxidase activity compared with the AIA group, suggesting that the treatment activates antioxidant activity in the synovial tissue and decreases oxidative stress and damage [89]. In a similar study also investigating the effects of resveratrol on autophagic cell death and angiogenic response in an acute AIA model (same treatment groups are employed), results have additionally shown significantly reduced levels of IL-1b, CRP, and PGE_2_, which were also correlated to p62 expression (significantly inhibited by treatment as observed in the previous study) [90].

##### Silibinin (Flavonolignans)

**Figure 2:**
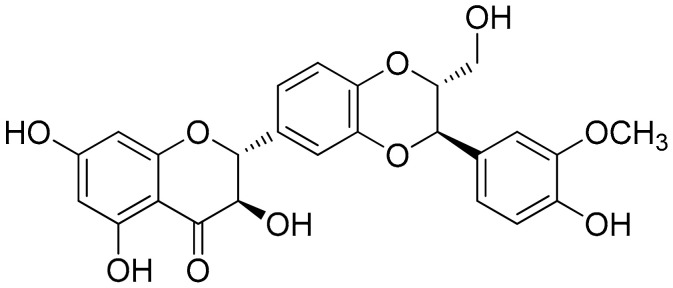
Silibinin structure.

Silibinin (Figure 2) is a natural polyphenolic flavonoid, with well-documented antioxidant, anti-inflammation, and anticancer properties [91]. A study showed that silibinin suppressed cell viability, suppressed the NF-κB pathway, decreased Sirtuin1, and increased the percentage of apoptotic RA-FLS, while RA-FLS transfection with a short hairpin RNA (shRNA) of SIRT1 enhanced silibinin-induced apoptosis. Notably, the selected concentrations of 0, 50, 100, and 200 μM of silibinin (cells treated for 48 h) dose-dependently induced the apoptosis of RA-FLS cells (apoptosis rates were approximately increased by 5% from 50 to 100 μΜ and from 100 to 200 μΜ), while in a similar way the same selected doses inhibited TNF-α-induced IL-6 and IL-1β production. It is noteworthy that further Western plot analysis toward these findings indicated that TNF-α-induced phosphorylation of NF-κB p65 and IκBα was also suppressed by silibinin in a concentration-dependent manner. Furthermore, as has also been investigated for RA-FLS cells, the combined effect of SIRT1-shRNA the treatment seemed to have higher outcomes (almost 30%) regarding the apoptosis rate compared to each treatment alone (apoptosis rate for silibinin 100 μΜ or SIRT1-shRNA were approximately 10%–15%). Similar results were observed in the setting of a CIA rat model, where 50, 100, and 150 mg/kg of silibinin were the selected doses of treatment. In these conditions, all treatments improved the arthritis score (especially after 8 days of treatment) with small variations reported regarding dose-dependency. Finally, TNF-α, IL-1β, and IL-6 levels in the treated group were also significantly decreased, as compared to the untreated group, in a dose-dependent manner [92].

##### Curcumin (Flavonoids)

**Figure 3:**
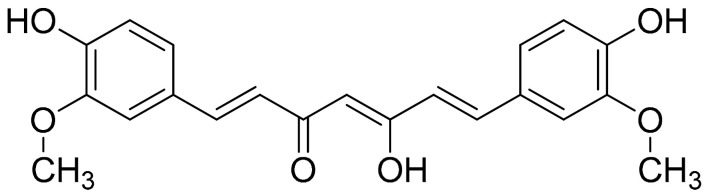
Curcumin structure.

Curcumin (Figure 3) is a well-studied polyphenol and the predominant active compound found in turmeric (*Curcumina longa*); although it is described as a compound with low bioavailability due to its fast metabolism in the liver (an event that has been previously described to be partially modulated via the coadministration of piperine), it was found to inhibit the expression of proinflammatory cytokines and chemokines, through suppression of the NF-κB signaling pathway [93]. It should be noted that a previous study investigating *Curcuma longa* (among other plants) has demonstrated that Curcumin Monoglucoside and Curcumin Diglucoside, exhibited strong molecular interactions at the potential ligand binding sites of TNF-α and IL-1, thus supporting the inhibitory effects against the proinflammatory cytokines TNF-α and IL-1 [60]. A recent study investigated cell viability (CCK-8 assay) and macrophage apoptotic effect of curcumin (flow cytometry, TUNEL assay), showing that curcumin inhibited the degradation of IκBα and reduced the production of COX-2 in LPS-induced inflammatory RAW264.7 cells. This research concludes that curcumin significantly induced macrophage apoptosis, presumably via the inhibition of the NF-κB signaling pathway [94]. The same study also investigated the therapeutic effects of orally administered curcumin versus MTX (0.3 mg/kg) on CIA rats. The intervention (200 and 100 mg/kg) provided daily (for approximately 1 week after CIA induction) was able to attenuate the degree of joint swelling, while the higher dose, as well as MTX, seemed to have better results regarding the evaluation of arthritis score, synovial hyperplasia score, and pannus formation score as compared to the higher dose of treatment. Finally, the increased levels of NF-α, IL-17, IL-1β, and TGF-β in CIA rat synovium were significantly inhibited by the treatment with 200 mg/kg curcumin or MTX (*p* < 0.05). Interestingly, although the high-dose treatment and MTX were closer together, regarding proinflammatory cytokines modulation, the effects of the lower dose were not far behind compared to those of the high dose, especially in terms of TNF-α and IL-17 levels [94]. 

##### Morin (Flavonoids)

**Figure 4:**
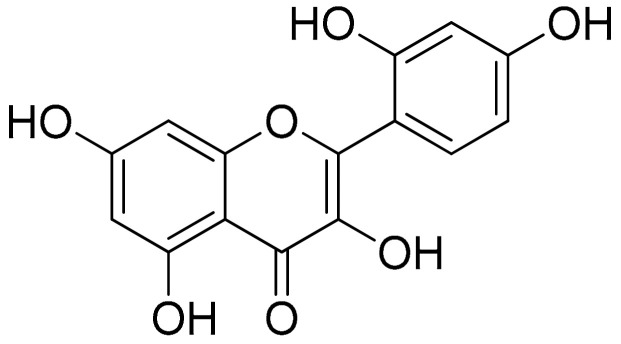
Morin structure.

This pentahydroxy flavone is a structural derivative of 1-benzopyran phenylpropanoid, commonly isolated from *Maclura pomifera* (Osage orange) and *Psidium guajava* (common guava) leaves, and has been previously related to allergic response, although as part of the flavonoid family it has also been reported to have antioxidant, antimicrobial, and anti-inflammatory properties [95,96,97]. An interesting study was conducted in an effort to evaluate the potential effects of morin (Figure 4) (30 mg/kg b.wt.) compared to a common therapeutic approach of indomethacin (3 mg/kg b.wt.) and also to explore any added value of their combination in the setting of CFA-stimulated Wistar albino rats. After almost 3 weeks of investigation (all treatments were administered intraperitoneally for 10 days—from day 11 to 20 after administration of CFA), this study demonstrated that the combined treatment was able to effectively regulate TNF-α (0.181 ± 0.0116, 0.149 ± 0.0069, 0.137 ± 0.0054 and 0.125 ± 0.0042, for the arthritic control group, morin group, indomethacin group, and combination group, respectively) and IL-1β (0.366 ± 0.0127, 0.143 ± 0.0190, 0.242 ± 0.0103 and 0.106 ± 0.007, for the respected groups). Similar results were reported regarding the levels of IL-17, IL-6, MCP-1 (0.247 ± 0.0142, 0.145 ± 0.01, 0.115 ± 0.0068, and 0.096 ± 0.0058 for the arthritic control group, morin group, indomethacin group, and combination group, respectively), and PGE_2_ in the serum of adjuvant-induced arthritic rats. Modulations were also reported for the RANKL and the transcription factors NF-κB p65 and AP-1 as combination treatments had lower RANKL and NF-κB p65 expression folds as compared to the indomethacin treatment but not compared to the morin treatment. Furthermore, morin and indomethacin cotreatment in arthritic rats demonstrated reduced paw edema, bone collagen levels, cartilage erosion, and synovial hyperplasia in addition to low to moderate inflammation scores (as described in the respected publication on a 0 to 3 scale) compared to the AIA control group’s severe score [98].

Morin was further studied for its antioxidant properties evaluating lipid peroxidation (nm of malondialdehyde formed/mg protein) and NO (μm of H_2_O_2_ consumed/min/mg protein) in the joint homogenate in the respected evaluation groups of the animals (control, arthritic rats, arthritic rats treated with morin, arthritic rats treated with indomethacin, arthritic rats treated with both morin and indomethacin, and control treated with morin). The experimental results demonstrated a reduction in all treatment groups as compared to the untreated arthritic rats in both the lipid peroxidation and NO (arthritic rats: lipid peroxidation, 9.40 ± 0.12, NO 9.29 ± 0.16; arthritic rats + morin (30 mg/kg b.wt.): lipid peroxidation, 7.25 ± 0.21, NO 7.37 ± 0.26, arthritic rats + indomethacin (3 mg/kg b.wt.): lipid peroxidation, 7.40 ± 0.25, NO 6.58 ± 0.28). However, the reduction was even greater in the cotreatment group (arthritic rats + morin (30 mg/kg b.wt.) + indomethacin (3 mg/kg b.wt.): lipid peroxidation, 6.68 ± 0.31, NO 5.56 ± 0.11), while treatment with morin was also able to lower the levels of lipid peroxidation and NO in the control group (control + morin (30 mg/kg b.wt.): lipid peroxidation, 6.51 ± 0.26, NO 5.39 ± 0.29) [98]. These results highlight both the potential of natural products as complementary treatments as well as the added value of antioxidants in the management of inflammation in RA.

##### Epicatechin-3-*O*-β-d-Allopyranoside (Flavonoids)

In a similar context, epicatechin-3-*O*-β-d-allopyranoside, is a flavan-3-ol isolated from *Davallia formosana*. This flavonoid was investigated in a recent study regarding its antiarthritic and anti-inflammatory effects. The study reports that in the CIA mice model, both treatments of 50 and 100 mg/kg epicatechin-3-*O*-β-d-allopyranoside dose-dependently suppressed arthritic symptoms after one month of daily supplementation (4.8 ± 0.8 and 3.3 ± 0.9, respectively, compared to the mean arthritis severity score of 6.6 ± 1.3 in the control group treated with water). Furthermore, in this study, it is reported that the supplementation was able to downregulate the production of IL-17 and TNF-α and increase the levels of IL-10 and IL-4 in the treatment groups, while IFN-γ rates were not affected by the treatment. Remarkably, dose-dependency in the rates of cytokine production was more obvious in the cases of TNF-α and IL-10 [99]. 

#### 3.2.2. Alkaloids

##### Sinomenine (Morphine Alkaloid)

**Figure 5:**
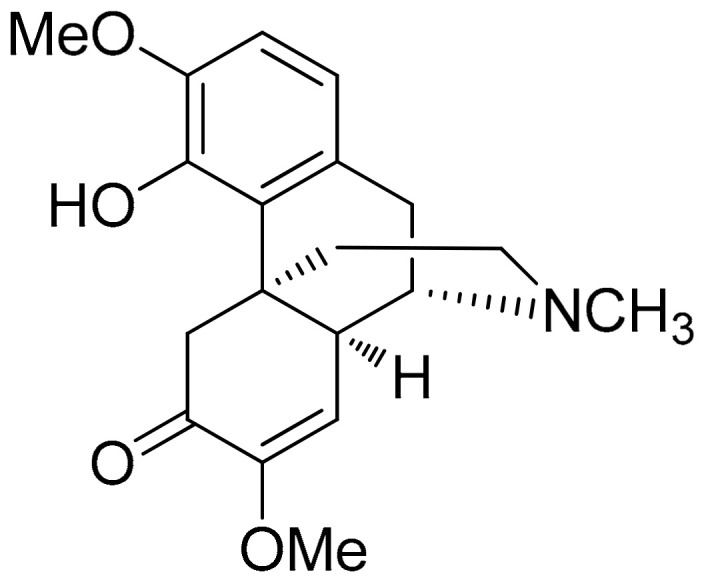
Sinomenine structure.

Sinomenine or cocculine (Figure 5) is a well-known morphinane alkaloid (morphinan-derived alkaloid) commonly found in the root of *Sinomenium acutum* (native to Japan and China). This plant is traditionally known in herbal medicine for over 2000 years and has been utilized in the treatment of RA in China [100]. Although Sinomenine is a prescription drug for RA in China, a recent study was conducted to further evaluate and analyze its effect in the setting of LPS-stimulated RAW264.7 cells via measuring the expression of cytokines and chemokines related to inflammatory progression. In this research, concentrations of 0–1000 μg/mL Sinomenine were tested and those of 10 and 50 µg/mL of Sinomenine was selected based on the cytotoxicity screening previously conducted via the cell counting kit-8 (CCK-8) assay. In particular, the 24 h treatment with Sinomenine 0.1–50 μg/mL resulted in almost 100% cell viability. LPS treatment was tested in various concentrations as well (0.01–20 μg/mL), which showed a gradual reduction in cell viability after the concentration of 1 μg/mL in the 24 h treatment (2 h preincubation time). In a more targeted approach, dose-dependent reductions in the secretion of IL-6, GMC-SF, IL-1a, IL-1b, TNF-α, and Eotaxin-2, were also observed in LPS-induced RAW264.7 model (1 μg/mL) cells. This study was carried further in CIA DBA/1 mice (groups: placebo, 50 or 100 mg/kg/day treatments). Lower inflammatory cell infiltration and synovial hyperplasia were demonstrated in addition to ameliorated clinical arthritis scores, while paw swelling score, inflammation score, and cartilage damage score were also decreased in a dose-dependent manner [101]. 

#### 3.2.3. Terpenes

##### Taraxasterol (Pentacyclic Triterpene)

*Taraxacum officinale* has been previously investigated in various settings regarding its bioactive properties including those against inflammation [102,103,104]. 

**Figure 6:**
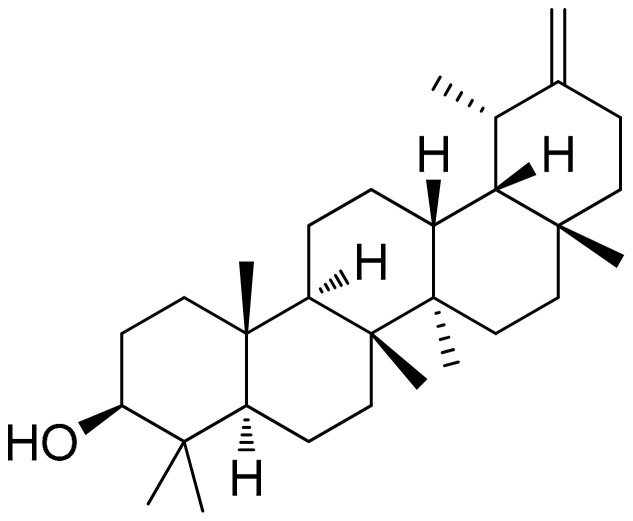
Taraxasterol structure.

A recent study investigated taraxasterol (Figure 6), a pentacyclic-triterpene derived from taraxastane (mevalonate pathway), found in *Taraxacum officinale* of Chinese origin, in IL-1β-stimulated (10 ng/mL) human RA-FLS in vitro as well in a CIA model in mice (CIA was induced by two immunizations in mice (secondary immunization was given 12 days after the primary immunization)). Results of this study as related to the in vitro model report that cell viability was not affected by the treatment while the selected doses (0.3 to 30 μM) showed no inhibition on IL-1β-induced proliferation of human RA-FLS. Additionally, also regarding the in vitro model, the anti-inflammatory activity of the treatment was detected based on significant suppressions of TNF-α, IL-6, and IL-8 (mostly observed in the doses of 3, 10, and 30 μM) and production of matrix metalloproteinases MMP-1 and MMP-3. Notably, the supplementation at a high dose (30 μM) significantly blocked the IL-1β-mediated NF-κB p65 nuclear translocation. The same study, investigating CIA mice, reported that the treatment (10 mg/kg, intragastrical administration every other day from day 0 to day 48), significantly decreased the expression of TNF-α, IL-6, and IL-8 in joint tissues (*p* < 0.01, compared with the vehicle-treated group) in addition to the modulation of CIA-induced levels of IKKα/β and IκBα phosphorylation and IκBα degradation [105].

Similar effects on the TNF-α and IL-6 levels were observed in another study investigating a different cell model (RAW264.7 macrophage cells). As documented in this case, the supplementation with the ethyl acetate fraction from *Taraxacum coreanum*, at doses of 5, 25, 50, and 100 μg/mL presented no cytotoxic effects while leading to significant and dose-dependent reductions in TNF-α and IL-6 in lipopolysaccharide (LPS)/interferon-gamma (IFN-γ)-induced RAW264.7 cells. It must be noted that this study addressed luteolin and luteoloside as the compounds responsible for these effects as they have been investigated at doses of 0.5, 2.5, 5, and 10 μg/mL, presenting similar effects as those of the ethanolic extract regarding the modulation of TNF-α and IL-6 levels in a dose-dependent manner [106].

##### Betulinic Acid (Pentacyclic Triterpene)

**Figure 7:**
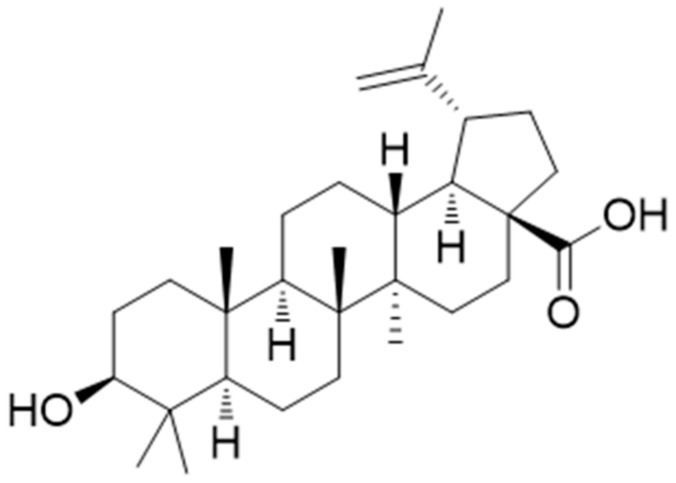
Betulinic acid structure.

Betulinic acid (Figure 7) is a naturally occurring pentacyclic triterpenoid. A study demonstrated that betulinic acid treatment (at concentrations of 0, 2.5, 5, and 10 mM) concentration-dependently suppressed the migration, invasion, and reorganization of the actin cytoskeleton of RA-FLSs. Additionally, in the setting of TNF-α-induced RA-FLSs, the treatment markedly downregulated the mRNA expression of IL-1β (no dose-dependency observed in the expression levels), IL-6, IL-8 (dose-dependent fold change was reported for both IL-6 and IL-8) and IL-17A (greater fold changed in the expression levels observed at the higher doses of 5 and 10 μΜ). Similarly, the treatment was also able to reduce the TNF-α-induced activation of the NF-κB signal pathway and the NF-κB nuclear accumulation (relative expression levels of phosphorylated NF-κB, IκBα, and IKK were dose-dependently decreased as compared to the untreated cells). Additionally, the investigation of betulinic acid in CIA mice showed that the treatment (20 mg/kg/day for 3 weeks from arthritis onset) was able to reduce arthritis score and paw swelling, with results of significantly reduced scores being noted toward the end of the second week onward [107].

##### Madecassoside (Pentacyclic Triterpene)

**Figure 8:**
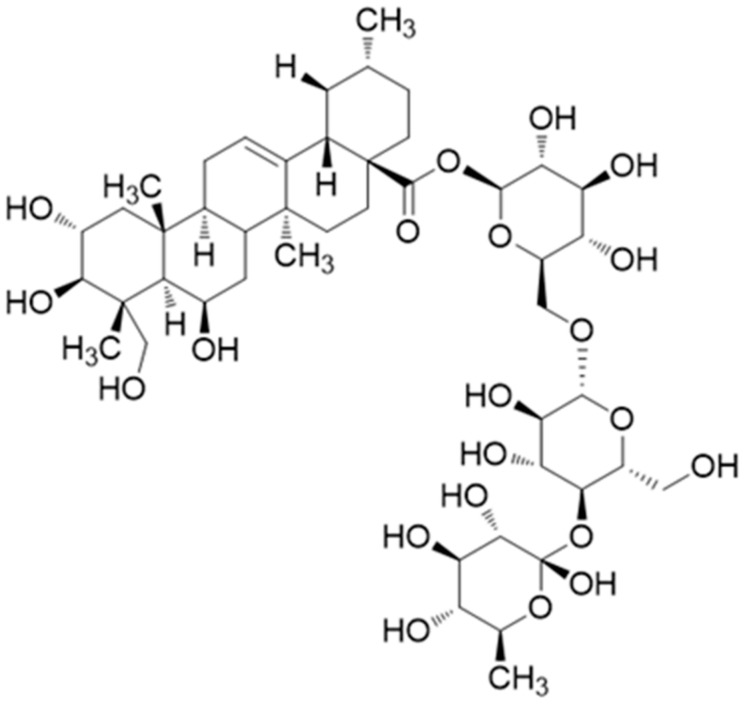
Madecassoside structure.

Madecassoside (Figure 8), a triterpenoid saponin present in *Centella asiatica* herbs, exerted an obvious therapeutic effect, as reported in a recent study, ameliorating the histological lesions in adjuvant-induced arthritis (AIA) rats. The main observations of 25 mg/kg supplementation with madecassoside (Figure 7) on body weight loss, polyarthritis index score, and reduction in paw swelling were overall improved compared to the untreated group. Compared to dexamethasone (0.5 mg/gr), the outcomes of madecassoside supplementation regarding paw swelling were slightly lower, although no statistical significance was documented. In a more targeted approach toward the antirheumatoid potentials of this compound (10 and 30 μmol/L), its interference with IL-1β-induced FLS cell invasion and MMP expression was investigated. Results of this study showed that the selected treatment concentrations effectively inhibited the migration and invasion (via modulating the expression of MMP-13) of IL-1β-induced FLS without exhibiting any effect on cell proliferation and cell viability (MTT assay).

Another interesting observation was that the mRNA expressions of MMP-2, MMP-3, and MMP-9 were affected by the treatment; however, no dose-dependency was observed in the outcomes, while the expression of MMP-13 was reported to have a notable fold chance, which was related to the treatment dose. An interesting finding in relation to this is that although the expression of MMP-13 was reduced in a dose-dependent manner, the results evaluating its enzymic activity did not follow up in the same way. Additionally, the treatment with madecassoside also downregulated the translocation and phosphorylation of NF-κB, suggesting an anti-RA activity related to the inhibition of the NF-κB/MMP-13 pathway [108].

##### Germacrone (Sesquiterpenoid)

**Figure 9:**
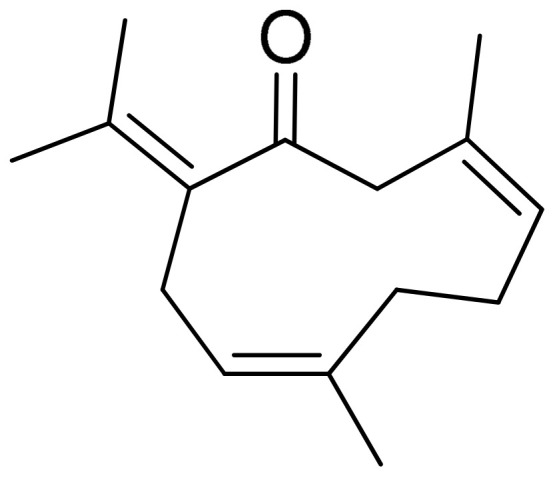
Germacrone structure.

Germacrone (Figure 9) is a phenol lipid of the class of sesquiterpenoids and another major bioactive derivative of *Rhizoma Curcuma*. In the setting of the CIA model of male DBA/1 J mice, germacrone treatment (orally administered 20 mg/kg daily, starting on day 21, following booster immunization conducted on the same day) significantly reduced arthritis score. Arthritis scores were almost cut in half in the treated mice as compared to the untreated CIA mice, especially after day 33. Furthermore, the levels of Th1 cytokines (TNF-α and IFN-γ) in mouse serum and synovial tissues were partially decreased (reduction levels were greater in the serum and almost decreased by 50% in the cases of TNF-α in serum and IFN-γ both in serum and synovial tissues). In a similar way, the Th2 cytokine (IL-4), which was initially reduced after the establishment of CIA, was increased by the treatment reaching close to yet not statistically significant levels as compared to the control group. Remarkably, IL-4 in serum was almost 12 pg/mL in the control group and nearly 10 pg/mL in the treated CIA mice, while IL-4 levels in the synovial tissues were approximately 160 pg/mL in the control group and nearly 140 pg/mL in the treatment group (CIA untreated values were less than 4 pg/mL and approximately 75 pg/mL in the serum and synovial tissues, respectively). In relation to this, it is also reported that the treatment decreased the ratio of Th1/Th2 cells and improved IκB expression, but suppressed p-p65 level in CIA mice which, overall, led to the interpretation that the alleviated progression of arthritis by the treatment might be related to the regulation of Th1/Th2 balance and inactivation of the NF-κB pathway [109].

##### Triptolide (Diterpene Triepoxide)

**Figure 10:**
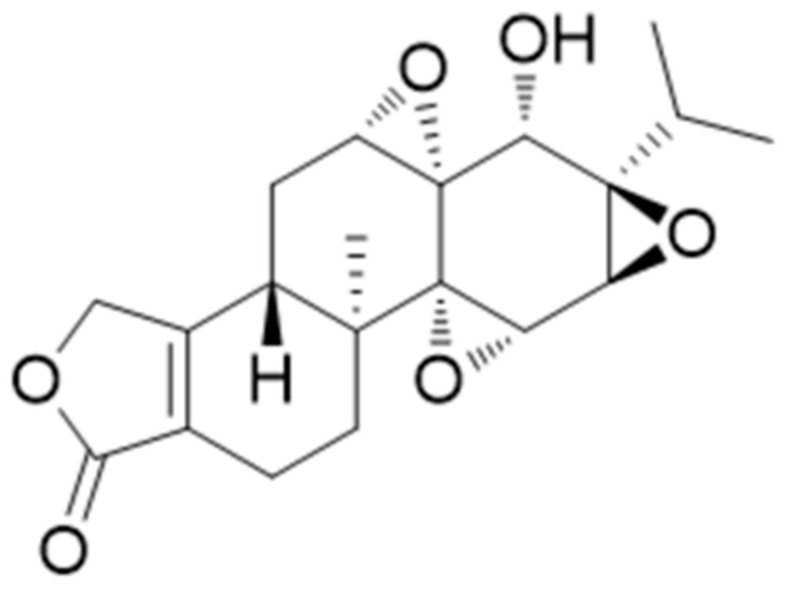
Triptolide structure.

Triptolide (TP) (Figure 10) is a diterpene triepoxide commonly found in *Tripterygium wilfordii* Hook F. A triptolide-loaded transdermal delivery system has been developed and evaluated in a study in a CIA rat model. The effects of triptolide were evaluated in doses of 10, 20, and 40 mg/kg, exhibiting that the treatment in all cases mitigated the degree of joint swelling, with more pronounced outcomes being observed in the high and medium doses, especially from the mid-third week of treatment onwards. Additionally, all treatments effectively reduced the serum levels of IL-1β and IL-6, in which case it was also demonstrated that high and medium doses had a better effect compared to the low dose, while regarding the levels of IL-6 no obvious differences in the reduction rate were observed between medium- and high-dose groups. The therapeutic mechanism of TP-LHP might be related to the balance between Th1 and Th2, as well as inhibition of the expression and biological effects of vascular endothelial growth factor [110]. 

#### 3.2.4. Benzenoids and Other Classes 

##### Allylpyrocatechol (Benzenoids–Phenol ester)

**Figure 11:**
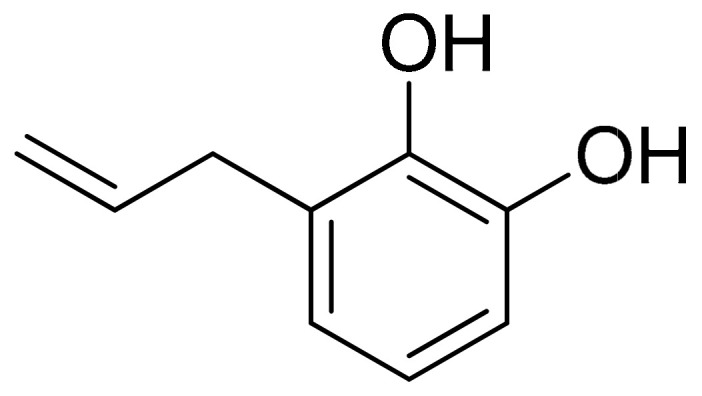
Allylpyrocatechol structure.

*Piper betle* (betel) plant belongs to the *Piperaceae* family, which includes pepper and kava. *Piper betle* is mostly consumed in Asia and its potent medicinal properties have already been documented, which might be mostly linked to some of its active compounds, such as piperol A, piperol B, allylpyrocatechol (Figure 11) (diacetate and monoacetate), ethylpiperbetol hydroxychavicol, piperbetol, eugenol, chavibetol, and alkaloids [111,112]. Allylpyrocatechol is an organic compound of the class of benzenoids, subclass of phenol esters. Evidence from a recent study support that allylpyrocatechol (at doses of 5, 10, or 20 mg/kg) was able to dose-dependently reduce paw edema and attenuate the damage to bones and cartilage degradation in an animal model established by immunizing rats with bovine collagen type II (CII) followed by lipopolysaccharide (LPS) (rats were immunized with CII emulsion on days 0 and 15, along with LPS (100 μg i.p.) on day 14). Interestingly, a dose-dependent reduction in plasma TNF-α levels was also observed after supplementation; however, no major alterations were observed in results regarding plasma IL-6 reduction concerning treatment dose [113]. Similar results were reported in research published in 2018 investigating the combined effect of allylpyrocatechol (20 mg/kg), and MTX (1.5 mg/kg) on limiting the progression of LPS accelerated CIA in Sprague–Dawley rats. The combined treatment resulted in the reduction in paw edema and inhibited the expression of proinflammatory cytokines, TNF-α, and IL-6. Furthermore, unlike MTX monotherapy, the combination treatment decreased the associated cachexia, splenomegaly, and oxidative stress [114].

##### Paeonol (Methoxybenzenes)

**Figure 12:**
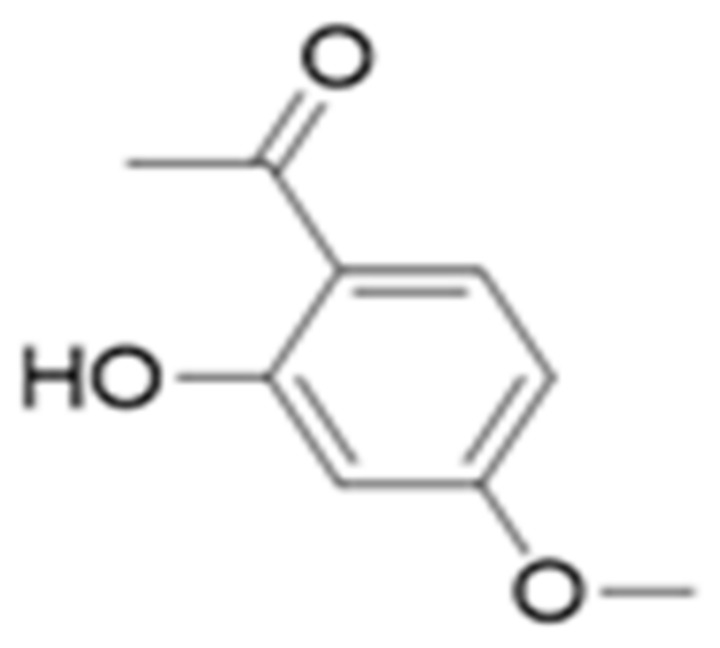
Paeonol structure.

This phenolic compound, also reported in the class of methoxybenzens, is commonly found in peonies and has been recently investigated in IL-1β-treated human RA-FLS. Although paeonol (Figure 12) treatment did not affect cell survival and IL-1β-induced (10 ng/mL) proliferation at doses of 0.1–100 μΜ (evaluation via the MTT assay), pretreatment with paeonol was able to successfully ameliorate the production of proinflammatory TNF-α (although a significant reduction was observed against the control group—*p* < 0.01 vs. IL-1β without paeonol—TNF-α levels were not affected by the treatment dose). In a similar way, the levels of IL-6, IL-1β, and the expressions of MMP-1/MMP-3 were also dose-dependently downregulated by the treatment (0.1, 1 or 10 μM paeonol) and TLR4 expression and NF-κB p65 activation were also inhibited (paeonol treatment strongly inhibited (*p* < 0.01) both NF-κB p65 subunit phosphorylation and IκBα degradation) [115]. Furthermore, the treatment (10 mg/kg of paeonol administration every other day from day 10 to day 48) in CIA mice attenuated clinical arthritis scores at almost half the mean values after day 30 compared to the control group. 

In the same context, decreased (*p* < 0.01) levels of proinflammatory cytokines (TNF-α and IL-6 were reduced in more than half the values of the untreated group) were reported in the serum, in addition to significant reductions (*p* < 0.01 vs. CIA without paeonol) of the MMP-1 and MMP-3 production in the ankle joints of CIA mice. Based on the treatment’s anti-inflammatory effects in synovial tissues, significant reduction in TLR4, and inhibitory actions on RA progression (as it is also described via the evaluation of clinical scores), the study concludes that paeonol can be a potential agent against RA, underling that the potential mechanism of action might be based on the attenuation TLR4-NF-κB activation [115].

##### Brazilin (Benzopyrans)

**Figure 13:**
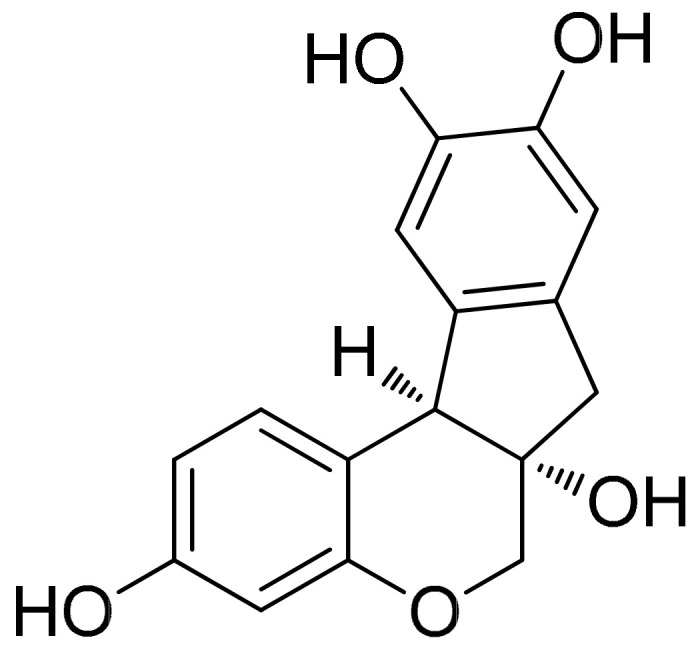
Brazilin structure.

Brazilin (Figure 13) is an organic heterotetracyclic compound of pyrans, a subclass of benzopyrans, and the major compound found in the *Caesalpinia sappan* L. (Leguminosae) and possesses proapoptotic and anti-inflammation potentials. Investigating its effects on RA-FLS, a study showed that the pretreatment with brazilin (25 μg/mL) was able to successfully reduce LPS-induced or TNF-induced NF-κB activation and the secretion of inflammatory cytokines in parallel with the enhanced autophagic flux. It is worth noting that when the cells were pretreated with brazilin (induction of autophagy evidenced by the conversion of LC3-II), LPS and TNF-induced NF-κB activation was drastically inhibited by the treatment, while under the condition of the blockade of the ROS-mediated autophagy pathway using NAC, the treatment failed to inhibit TNF-induced phosphorylation and degradation of IκBα [116].

##### Sulforaphane (Isothiocyanates)

**Figure 14:**
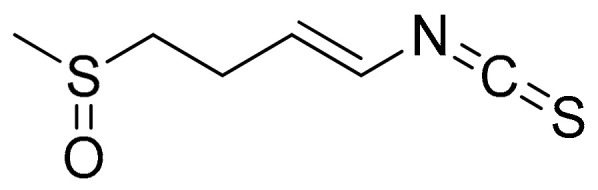
Sulforaphane structure.

Sulforaphane (Figure 14) is a widely investigated compound of the family of isothiocyanates (the products of degradation of glucosinolates) which are present in cruciferous vegetables, particularly broccoli and broccoli sprouts [117]. This compound, that acts via cytoplasmic Nuclear factor (erythroid-derived 2)-like 2 (Nrf2) to enhance the production of antioxidants in the brain through the glutathione pathway, has been the focus of accumulating research due to its promising health-promoting properties in disease and low toxicity in normal tissue [117,118]. It is noteworthy that relevant research also explores the potential of this compound as an inhibitor of the proinflammatory activity of the cytokine macrophage migration inhibitory factor (MIF). The mechanism described via the inactivation of tautomerasic activity is related to many chronic diseases and previous reports have demonstrated the potential of sulforaphane, benzyl, n-hexyl, and phenethyl isothiocyanates in this setting [119].

Research has shown that sulforaphane treatment (10 μΜ), in addition to decreased expression of the transcription factor RORγt, as well as IL-17A, IL-17F, and IL-22, also induces a pro-oxidative state in untransformed human T-cells of healthy donors or RA patients translated as an increase in intracellular reactive oxygen species (ROS) and a marked decrease in glutathione (GSH). These results highlight a potential role of natural products in balancing the T-cell redox milieu [120]. Additionally, previous evaluation of antiarthritic activity in FCA-induced arthritic rats (sulforaphane treatment: 5 mg/kg) has shown decreased production of TNF-α, IL-6, and INF-γ in addition to a significant reduction in synovial inflammatory infiltration [121]. Similar results were reported in a murine monoarthritis model (CFA-10 μg/joint, in 10 μL), where the animals received either sulforaphane (10 mg/kg) or vehicle, intraperitoneally (i.p.), twice a day for 3 days. Observations of this study also showed that the treatment reduced joint swelling and damage, presented higher levels of IL-6, and also increased the recruitment of lymphocyte antigen 6 (Ly6C+ and Ly6G+) cells to CFA-injected joints. It is noteworthy that the treated animals also presented downregulation of CD11b and CD62L on synovial fluid Ly6G+ cells as well as increased activity of TrxR (which has been correlated with disease risk in patients with RA), in comparison with controls [122,123].

In the setting of RA, a recent study on collagen-induced arthritis (CIA) mice investigated the effect of sulforaphane treatment (1.5 mM, 200 μL of sulforaphane at 12.8 mg/mL/kg) against a control group (phosphate buffered saline) over a 7-week treatment. Results of this study report an overall improvement in arthritis score as well as histological findings such as inflammation, cartilage damage, and bone erosion in the joint. Additionally, the anti-inflammatory effects of the treatment were associated with reduced expression of inflammatory cytokines in the joints of CIA mice (IL-6-, IL-17-, and TNF-α), which was reflected as a 50% reduction (levels in the culture supernatant were measured by ELISA) compared to the control group. A noteworthy finding of this research is that sulforaphane also decreased the differentiation of LPS-stimulated murine splenocytes into plasma B cells and germinal-center B cells, which was highlighted by the researchers as a potential antiarthritic mechanism of action. However, a significant finding of this study is also that sulforaphane attenuated the production of IL-6, TNF-α, IL-17, and pathologic IgG in human peripheral blood mononuclear cytokines (PBMCs) (pro- to anti-inflammatory cytokines produced by mitogen-stimulated PBMCs has been also studied as biomarkers of RA progression) [118,124].

## 4. Discussion

The management and prevention of RA remain essential fields of global research in all its levels of investigation. As presented in this review (Table 2 and Table 3 provide an overview of results), antiarthritic evidence of basic in vitro studies of natural products are mostly established based on proinflammatory cytokine modulation, which in some cases is also related to antioxidant activity, as well as the effects of the investigated product on the induction of apoptosis. At the cellular level, the activation of macrophages, neutrophil granulocytes amongst other immune cells by LPS during bacterial infection or by cytokines (IL-1, TNF-α, or IFN-γ) induces overexpression of iNOS and overproduction of NO. Nonsteroidal anti-inflammatory medicines can block iNOS and scavenge NO. In relation to this, the antioxidant properties of natural products are markedly related to their ability to modulate the levels of free radicals and thus provide complimentary assistance to the RA treatment via inhibiting the detrimental effects of excessive NO production in the human body. In this field of research, RAW264.7 and RA-FLS cells are those commonly employed to investigate anti-inflammatory activities for RA, for the most part, based on the outcomes of TNF-α and IL-1β or IL-6 levels, and in some cases MMPs, while few studies reach further in the evaluation of transcription factors, using other mediators such as IL-1, IL-8 etcetera as well as Th1/Th2 ratio inter alia. However, PBMCs, which have also emerged as a promising field of investigation for proinflammatory markers as well as markers of disease progression, are not that widely used. In a similar way, although MIF has been implicated in the pathogenesis of both experimental and human rheumatoid arthritis (including the cognitive decline that can be observed in patients with RA), few studies have worked toward this direction of evaluation for either natural products or compounds [125,126,127]. As previously mentioned, among the implicated factors of RA, immune cells (among the most evaluated of which are macrophages, neutrophils, T cells, and dendritic cells) working in combination with nonimmune cells such as fibroblasts and chondrocytes, in addition to the commonly studied inflammatory mediators of cytokines, proteases, chemokines, and autoantibodies, in the inflammatory processes target the cartilage and bone, leading to functional loss of joints. Further approaches consider the study of the implicated pathways as well as molecular binding in addition to several preparation methods and different doses that are usually considered in this stage, which also provide significant insights on the development of the hypothesis to be tested in the in vivo setting. Additionally, CIA and FCA-induced models are the main settings of investigation in rats or mice regarding RA. In both cases, the expression levels of proinflammatory cytocides in serum remain the first step of evaluation, followed by the assessment of paw or ear edema, hot-plate test for the study of pain reduction, and body-weight evaluation, which is also described as a good marker for the disease’s progression. 

Summarizing this review, it is clear that several natural products (plenty of which have origins in traditional medicine) have the potential to be utilized in the setting of RA. Regardless of the preparation method of the plant extracts, in most cases, basic parameters such as TNF-α and IL-6 reduction, indicative of anti-inflammatory activity, are effectively modulated; however, several considerations must be addressed on this topic, bearing in mind that the ultimate target is to translate these effects for humans. For instance, from a nutrition/diet perspective, the evaluation of natural products as aqueous extracts could provide insights for RA prevention as well as management, while the same could not apply for organic fractions of the aqueous extract regardless of its benefits. On the other hand, the evaluation of fractions, as well as the identification and isolation of pure compounds, is essential for the development of new drugs that will effectively target the disease modulators. Considering that research is designed to be conducted in very well-controlled conditions, inevitable real-world variations concerning both the human nature as well as the product or compound, such as genetics, cultivation conditions, effective extraction methods, bioavailability, age, sex, comorbidities, and many other diversities will require further and harder troubleshooting as we move from the in vitro outcomes to the animal models and then to clinical trials. Although in some cases, as presented by this review, the outcomes of basic research manage to be transferred in the clinical setting, the evidence is mostly based on results in either small patient groups or highly targeted patient groups (in terms of age, sex, menopausal status, comorbidities wtc.), while the number of such efforts is unable to create a solid case of the importance of natural products as a complementary treatment. This reflects the bottleneck seen in the research community hoping to move forward to the exploration and exploitation of green and sustainable medicinal research.

## 5. Conclusions

This study highlights the methods and the results of in vitro and animal in basic research of natural products in the management of RA over the past five years. It is important to mention that most of the natural products (mixtures or isolated compounds) provide a multitarget profile, which has to be considered for further utilization of natural products in the treatment of such chronic diseases.

## Figures and Tables

**Table 1:** Inclusion/exclusion criteria of the studied papers.

Criterion	Inclusion	Exclusion
Study Design	All relevant in vitro studies and animal studies	Reviews, clinical trials, case reports, editorials, letters, unpublished work, study protocols
Intervention *	Natural products or isolated compounds thereof	Common medication, acupuncture, massage, relaxation, and mind–body exercises
Comparator *	Nonsteroidal anti-inflammatory drugs (NSAIDs), Steroids (corticosteroid medications, such as prednisone), Disease-modifying antirheumatic drugs (DMARDs), Biologic agents	Acupuncture, massage, relaxation, and mind-body exercises alone or along with a natural product intervention
Outcomes *	Reduction in inflammation, pain and/or swelling relief	Any other outcomes that cannot support anti-inflammatory activity and/or pain and swelling relief
Language	English	Non-English full text available
Time Frame	2015 onwards	Earlier than 2015

* Applicable in animal models only.

**Table 2:** Natural products presented in this review as per study design and Rheumatoid arthritis (RA)-related effects reported.

Natural Product	Study Design	Comparator	Effects Described
*Punica granatum* Linn. ^#^	In vitro (LPS-induced RAW264.7)	N/A *	NO inhibition (tannin-related)
	In vivo (CIA mice—dose: 100 mg/kg b.wt.)	N/A *	Reduction in paw edema
	In vivo (FCA rats—doses: 50 or 75 mg/kg b.wt.)	Dexamethasone (5 mg/kg)	Improvement in arthritic score, body weight, paw volume and joint diameter
*Tripterygium wilfordii ^#^*	In vivo (CIA rats—doses: 2.5 or 5 mg/100 g b.wt. plant extract or 4 μg/100 g b.wt. isolated triptolide)	N/A *	Improvement in bone erosion, paw swelling and total protein concentration in the SFs Inhibition of the DPPI activity in the serum
*Schefflera octophylla* (Lour.) Harms	In vivo (mice—doses examined in the hot plate test: 300, 600 and 1200 mg/kg b.wt.)	N/A *	Improvement in the pain threshold
	In vivo (mice—doses examined in the xylene-induced ear edema test: 300, 600 and 1200 mg/kg b.wt.)	Indomethacin (10 mg/kg)	Reduced ear edema
	In vivo (FCA rats—doses: 150, 300 or 600 mg/kg of the ethanol extract or doses: 75, 150, and 300 mg/kg of the chloroform fraction)	Leflunomide (6 mg/kg)	Reduction in paw swelling and pain Improvement in body weight and arthritis score Downregulation of TNF-α, IL-1β and IL-6 expression levels
*Phoenix dactylifera* L.	In vitro	N/A *	Antioxidant activity (DPPH)
	In vivo (CIA rats—dose: 30 mg/kg b.wt.)	Indomethacin (10 mg/kg)	Reduction in paw and ear edema
*Ribes orientale*, *Ribes alpestre* Decne	In vivo (FCA rats—doses: 50, 100 or 200 mg/kg b.wt.)	Piroxicam (10 mg/kg)	Improvement in arthritis score and paw edema Downregulation of PGE_2_, COX-2, IL-1β IL-6, NF-κB and TNF-α expression levels Upregulation of IL-4, IL-10 expression levels
	In vivo (FCA rats—dose: 200 mg/kg b.wt.)	Piroxicam (10 mg/kg)	Reduction in paw volume and thickness Improvement in arthritic score Downregulation of IL-1β, TNF-α, IL-6, COX-2, PGE_2_ and NF-κB Antioxidant activity (DPPH, FRAP)
*Paullinia pinnata* L.	In vitro (LPS-induced RAW 264.7—doses: 10, 30 and 100 μg/mL)	N/A *	Reduction in NO, TNF-α and IL-1β production
	In vivo (FCA rats—doses: 100, 200 and 300 mg/kg)	Diclofenac (5 mg/kg)	Antioxidant properties (NO inhibition) Reduction in paw swelling and pain
*Camellia sinensis* (L.)	In vitro	N/A *	Inhibition of TNF-α and IL-1 (related to the component epigallocatechin-3-gallate)
	In vivo (CIA mice—dose: 400 mg/kg b.wt.)	N/A*	Alleviation of joint deformity, tissue swelling, pannus formation and neutrophils infiltration Antioxidant activity (NO inhibition)
*Smilax glabra*	In vitro (LPS-induced RAW 264.7)	N/A*	Reduction in TNF-α, and IL-6 expression levels Inhibition of ERK and JNK
	In vitro (LPS-induced THP-1)	N/A*	Reduction in IL-1 expression levels Antioxidant activity (NO inhibition, iNOS expression)
*Circaea mollis* Sieb. & Zucc	In vivo (FCA rats—doses: 170, 680 or 1350 mg/kg b.wt.)	Aspirin (35 mg/kg)	Improvement in arthritis score Reduction in paw swelling and pain score Downregulation of serum TNF-α and IL-1β Increase in serum IL-10 production
*Clematis orientalis* Linn	In vivo (FCA rats—dose: 200 mg/kg b.wt.)	Piroxicam (10 mg/kg)	Reduction in paw volume and joint thickness Downregulation of IL-1β, TNF-α, IL-6, COX-2 PGE2 and NF-κΒ expression levels
*Ephedra gerardiana*	In vivo (FCA rats—dose: 200 mg/kg b.wt.)	Piroxicam (10 mg/kg)	Reduction in paw swelling Improvement in arthritis score Downregulation of PGE_2_, IL-1β, IL-6, NF-κΒ and TNF-α expression levels
	In vitro (10μl of ethanol or butanol extract)	N/A *	Antioxidant activity (DPPH, NO)
*Bawei Longzuan*	In vivo (CIA rats—doses: 1.25, 2.5 and 5 g/kg b.wt.)	Dexamethasone (0.25 mg/kg)	Reduction in paw swelling and arthritis score Downregulation of IL-1β, TNF-α, IL-6 and IFN-γ serum levels
*Matteuccia struthiopteris*, *Osmunda japonica*, *Matteuccia orientalis*, *Pteridium aqui-linum*	In vitro (LPS-induced RAW264.7)	N/A *	Reduction in IL-1β Antioxidant properties (DPPH, TEAC, ABTS)
	Doses: 2.5, 5, 10, 20, 40, 80 and 160 mg/mL		
*Tamarix ramosissima* Ledeb	In vitro (RA-FLS)	N/A *	Inhibition of cellular apoptosis Improvement in the activated caspase-3/7 levels and sub-G1 fraction in the cell cycle
*Eupatorium japonicum* Thunb.	In vitro (RA-FLS—dose: 37.5 μg/mL)	N/A *	Induction of ROS-mediated apoptosis Reduction in IL-1β expression and MMP-9 transcription Inhibition of NF-κB and p38 activation

* N/A: Comparator was either not reported or not applicable for the setting of investigation. ^#^ Clinical trials identified in the context of RA.

**Table 3:** Natural compounds presented in this review as per study design and reported RA-related effects.

Natural Compound	Study Design	Comparator	Effects Described
Sulforaphane	In vitro (LPS-induced murine splenocytes, PBMCs)	N/A *	Decreased the differentiation of LPS-stimulated cells and germinal-center B cells Attenuated the production of IL-6, TNF-α, IL-17, and IgG in human PBMCs Concentration- and time-dependence of the inactivation of MIF tautomerase activity
	In vitro (RAW 264.7)—concentrations: 0.2–10 µM (0.1% acetonitrile)	N/A *	Reduction in inflammation, cartilage damage, and bone erosion in the joint Reductions in expression of IL-6-, IL-17-, and TNF-α in the joints
	In vivo (CIA mice—dose: 12.8 mg/mL/kg)	N/A *	Decreased production of TNF-α, IL-6, INF-γ Reduction in synovial inflammatory infiltration
	In vivo (FCA rats—dose: 5 mg/kg)	N/A *	Reduced joint swelling and damage Increased levels of IL-6, and recruitment of Ly6C+ and Ly6G+ Down-regulation of CD11b and CD62L on synovial fluid Ly6G+
	In vitro (FCA-mice—dose: 10 mg/kg)	N/A *	Increased activity of TrxR
Sinomenine	In vitro (LPS-stimulated RAW264.7)	N/A *	Reduction in the secretion of IL-6, GMCSF, IL-1a, IL-1b, TNF-α, and Eotaxin-2
	In vivo (CIA mice—doses: 50 or 100 mg/kg)	N/A *	Reduction in inflammatory cell infiltration and synovial hyperplasia Reduction in arthritis scores, paw swelling and cartilage damage
Taraxasterol	In vitro (IL-1β-stimulated RA-FLS—doses: 0.3 to 30μM)	N/A *	Suppression of TNF-α, IL-6, and IL-8 Reduced production of MMP-1 and MMP-3 Inhibition of the IL-1β-mediated NF-κB p65 nuclear translocation
	In vivo (CIA mice—dose: 10mg/kg)	N/A *	Reduction in TNF-α, IL-6 and IL-8 expression in joint tissues Modulation of IKKα/β and IκBα phosphorylation and IκBα degradation
	In vitro (LPS-induced RAW264.7—doses: 5, 25, 50, and 100 μg/mL)	N/A *	Reduction in TNF-α and IL-6 levels
Curcumin	In vitro (LPS-induced RAW264.7)	N/A *	Inhibition of the degradation of IκBα Reduction in COX-2 production Induction of macrophage apoptosis
	In vivo (CIA rats—doses: 100 or 200 mg/kg)	MTX (0.3 mg/kg)	Reduction in joint swelling, arthritis score, synovial hyperplasia score, and pannus formation score Modulation of TNF-α, IL-17, IL-1β and TGF-β levels in CIA rat synovium
Morin	In vivo (CIA rats—dose: 30 mg/kg b.wt.)	Indomethacin (3 mg/kg)	Reduction in TNF-α, IL-1β, IL-17, IL-6, MCP-1, and PGE2 in serum Modulation of RANKL, and transcription factors NF-κB p65 and AP-1 Improvement in paw edema, bone collagen levels, cartilage erosion and synovial hyperplasia Inhibition of iNOS Reduction in Lipid peroxidation and NO levels
		Combination therapy (indomethacin + morin)	
Resveratrol	In vivo (AIA rats—dose: 12.5 mg/kg)	N/A *	Reduction in knee swelling Reduction in the histological score of synovial tissue Improvement in the expression of LC3 signals Mitigation of the p65 expression Reduction in articular cartilage degradation Reduction in IL-1β, CRP and PGE_2_ levels
Allylpyrocatechol	In vivo (CIA rats—doses: 5, 10, or 20 mg/kg)	N/A *	Reduction in paw edema, bone damage and cartilage degradation Reduction in plasma TNF-α and IL-6 levels Reduction in paw edema Inhibition of TNF-α, and IL-6 expression Diminish cachexia, splenomegaly, and oxidative stress
	In vivo (CIA rats—dose: 20 mg/kg)	MTX (1.5 mg/kg) Combination therapy	
Epicatechin-3-*O*-β-d-allopyranoside	In vivo (CIA rats—doses: 50 or 100 mg/kg)	N/A *	Suppression of arthritis symptoms and improvement in disease severity Downregulation of IL-17 and TNF-α levels Improvement in IL-10 and IL-4 levels
Paeonol	In vitro (IL-1β-stimulated RA-FLS—dose: 0.1–100μΜ)	N/A *	Reduction in TNF-α, IL-6, IL-1β, and the expressions of MMP-1/MMP-3 Inhibition of TLR4 expression and NF-κB p65 activation
	In vivo (CIA mice—dose: 10 mg/kg)	N/A *	Improvement in clinical arthritis scores Reduction in TNF-α, IL-6, MMP-1 and MMP-3 production in the ankle joints Antioxidant activity
Madecassoside	In vivo (AIA rats—dose: 25 mg/kg)	Dexamethasone (0.5 mg/gr)	Modulation of body weight loss, polyarthritis index score Reduction in paw swelling
	In vitro (IL-1β-stimulated RA-FLS—doses: 10 or 30 μmol/l)	N/A *	Inhibition of the migration and invasion (via modulating the expression of MMP-13) of IL-1β-induced FLS Modulation of the mRNA expression levels of MMP-2, MMP-3, MMP-9 and MMP-13 Downregulation of the translocation and phosphorylation of NF-κB
Silibinin	In vitro (RA-FLS—doses: 0, 50, 100, and 200 μM)	N/A *	Suppression of cell viability and NF-κB pathway Reduction in Sirtuin1 Improvement in the apoptotic RA-FLS Inhibition of the TNF-α-induced IL-6 and IL-1β production and phosphorylation of NF-κB p65 and IκBα
	In vivo (CIA rats—doses: 50, 100 and 150 mg/kg)	N/A *	Improvement in arthritis score Reduction in TNF-α, IL-1β and IL-6 levels Antioxidant properties
Brazilin	In vitro (RA-FLS—dose: 25μg/mL)	N/A *	Reduction in LPS-induced or TNF-induced NF-κB activation and the secretion of inflammatory cytokines
Germacrone	In vivo (CIA mice—dose: 20 mg/kg)	N/A *	Reduction in arthritis score Reduction in TNF-α and IFN-γ levels in serum and synovial tissues Improvement in IL-4 levels Reduction in the Th1/Th2 ratio Improvement in IκB expression Antioxidant activity
Betulinic acid	In vitro (RA-FLS—doses: of 0, 2.5, 5, and 10 mM)	N/A *	Inhibition of the migration, invasion and reorganization of the actin cytoskeleton of RA-FLS Downregulation of the mRNA expression of IL-1β, IL-6, IL-8 and IL-17A
	In vivo (CIA mice—dose: 20 mg/kg)	N/A *	Reduction in the TNF-α-induced activation of NF-κB signal pathway and the NF-κB nuclear accumulation Reduction in arthritis score and paw swelling
Triptolide	In vivo (CIA rats—doses: 10, 20 or 40 mg/kg)	N/A *	Reduction in joint swelling Reduction in IL-1β and IL-6 serum levels

* N/A: Comparator was either not reported or not applicable for the setting of investigation.

## Abbreviations

b.wt.	Body weight
DPPH	1:1-diphenyl-1-picryl-hydrazyl
MTT	3-(4,5-dimethylthiazol-2-yl)-2,5-diphenyltetrazolium bromide
AIA	Antigen-induced arthritis
CCK-8	Cell counting kit-8
JNK	c-Jun NH2-terminal kinase
CIA	Collagen-induced arthritis
Cs	Conventional synthetic
CRP	C-Reactive protein
COX	Cyclooxygenase
DPPI	Dipeptidyl peptidase I
DMARDs	Disease-modifying antirheumatic drugs
ESR	Erythrocyte Sedimentation Rate
ERK	Extracellular signal-regulated kinase
FRAP	Ferric reducing antioxidant power
FLS	Fibroblast-like synoviocytes
Foxp3	Forkhead box P3
FCA	Freund’s complete adjuvant
GMC-SF	Granulocyte-macrophage colony-stimulating factor
Hb	Hemoglobin
iNOS	Inducible NO synthetase
IRF-4	Interferon Regulatory Factor 4
IL	Interleukin
LPS	Lipopolysaccharide
MMP	Matrix metalloproteinases
MTX	Methotrexate
MARK	Mitogen-activated protein kinase
NSAIDs	Nonsteroidal anti-inflammatory drugs
NO	Nitric oxide
NF-κB	Nuclear factor kappa-light-chain-enhancer of activated B cells
Nrf2	Nuclear factor (erythroid-derived 2)-like 2
PBMCs	Peripheral blood mononuclear cytokines
PGE_2_	Prostaglandin E2
RORγt	RAR-related orphan receptor gamma
ROS	Reactive oxygen species
RANKL	Receptor activator of nuclear factor kappa-B ligand
Treg	Regulatory T cells
RA	Rheumatoid arthritis
RF	Rheumatoid factor
shRNA	Short hairpin RNA
STAT3	Signal transducer and activator of transcription 3
SEM	Standard error of the mean
SF	Synovial fluid
Th	T helper
TUNEL assay	Terminal deoxynucleotidyl transferase dUTP nick end labeling assay
TCM	Traditional Chinese medicine
TGF-β	Transforming growth factor-β
TEAC	Trolox equivalent antioxidant capacity
TNF	Tumor necrosis factor
